# Parents' Education Level and Mortality and Morbidity of Children after Liver Transplantation

**Published:** 2015-02-01

**Authors:** Z. Bahador, S. M. Dehghani, A. Bahador, S. Nikeghbalian, N. Hafezi, M. Bahador, S. A. Malek-Hosseini

**Affiliations:** 1 Medical School, Shiraz University of Medical Sciences, Shiraz, Iran; 2 Gastroenterohepatology Research Center, Shiraz Transplant Research Center, Shiraz University of Medical Sciences, Shiraz, Iran; 3 *Shiraz Organ Transplant Center, Nemazee Hospital, Shiraz University of Medical Sciences, Shiraz, Iran*

**Keywords:** Pediatrics, Liver transplantation, Postoperative complications, Educational status, Parents, Medication adherence

## Abstract

Background: So far numerous post-transplant outcome predictors have been studied to decrease the loss of resources and grafts after organ transplantation. The role of education, as a predictor, in liver transplantation outcome has so far been studied in several articles. However, in most of the studies it was evaluated as a surrogate for socioeconomic status or other variants. The absolute impact of parents’ education has rarely been studied. Adult patients are their own caregivers whereas pediatric liver transplantation recipients are mostly cared by their parents.

Objective: To evaluate the effect of level of patients' education on the mortality and morbidity of pediatric liver transplant recipients.

Methods: We studied a group of 91 children who had undergone liver transplantation in our center from March 21, 2012 to July 21, 2013. In this retrospective study, patients’ medical charts and questionnaire were used to collect the necessary data. Post-transplantation mortality and complications were divided into two categories: Early (<6 months after liver transplantation), and late (≥6 months after the transplantation). Parents’ educational level was also categorized into 5 groups.

Results: Multivariate analysis of all groups showed that paternal education is an independent predictor of the late post-transplantation complications (p=0.024). Educational level of children’s mothers had no significant correlation with the late post-transplantation complications (p=0.45). Neither maternal (p=0.59) nor paternal (p=0.607) education had significant effect on the late post-transplantation mortality.

Conclusion: Paternal educational level of liver transplanted children is associated with the late post-transplantation complications.

## INTRODUCTION

Hepatic failure is one of the most common causes of death in children. Liver transplantation is a life-saving procedure for patients with end-stage liver disease (ESLD) that has become the standard of care for these patients since last decades [1]. Shortage of organ pool and high cost of this procedure pushed the liver transplantation centers to choose the best organ and recipient and consider the patient care givers (parents in children). This can help to decrease the complications, graft loss, mortality and waste of resources [1, 2].

Choosing a suitable treatment is not the only factor for the success of the therapy; the cooperation of patients also plays an important role [3]. In liver transplantation, post-transplantation compliance with medication is among the important factors that predicts graft survival [4-7].

Numerous post-transplantation outcome predictors have so far been studied to decrease the loss of resources and graft since the innovation of organs transplantation. The relationship between medication adherence (compliance) with factors such as low support, race, personality trait, religion, income, age, lack of partnership, and patient education have been studied in recent years. However, all of the above-mentioned factors have been studied in adult patients, not in children. Undoubtedly, the outcome of liver transplantation in children depends on their caregivers.

Considering that nearly all of the follow ups of young recipients are performed by their parents in our center, we conducted this study to determine if the level of education of parents of our pediatric recipients affects the mortality and morbidity of these patients.

## PATIENTS AND METHODS

In this study, 112 patients aged <18 years who underwent liver transplantation between March 21, 2012 and July 21, 2013 in Namazee Transplant Center, Shiraz, Iran were studied. The patients and/or their caregivers received preliminary medical training for medication taking, hygiene, medical follow-up, and red flags of health status at the time of discharge. The patients were weekly visited by the medical team of organ transplantation during the first month after discharging from the hospital. Thereafter, clinical complaints, laboratory data, and radiological imaging of patients were followed monthly by trained staff of the coordination office at the transplant center, and reported to the transplant team hepatologist. Every patient was called for visit if any problem was detected by the physician. 

We reviewed patients’ medical charts and used a questionnaire to collect the necessary data including demographic information, cause of cirrhosis, PELD and Child-Pugh score, graft type, immunosuppressive medication and its blood level, post-transplantation complications, and mortality, graft type, donor age, and parents’ education. 

Post-transplantation complications were categorized as “Early:” complications occurring before six months post-transplantation (*eg*, rejection, hepatic artery thrombosis, biliary complications, infection, convulsion, renal problems and cardiopulmonary complications), and “Late:” complications occurring after six months of transplantation. Since the complications occurring during the first six months of transplantation seems to be linked with the procedure and graft quality [2], the patients who were expired in the first six months were excluded from the study. Late complications consisted of vascular thrombosis, rejection, biliary obstruction, infections, drug side effects, PTLD, lymphoma and leukemia. For the purpose of analysis, parents’ education level was divided into five groups: 1) Illiterate; 2) <8 years; 3) between 8 and 12 years; 4) education between diploma and bachelor; and 5) higher education.

Patients who underwent multiple liver transplantation (n=4), those whose survival was less than six months (n=14), patients under supervision of a single parent (n=2), and those who did not provide reliable information (n=1) were excluded from the study.

## RESULTS

Ninety-one patients were included in this study. The mean±SD age of the patients at the time of transplantation was 8.0±5.8 (range: 0.5–18) years. Forty four (48%) patients were male. The patients had a mean weight of 26.46 kg at the time of transplantation.

**Figure 1:**
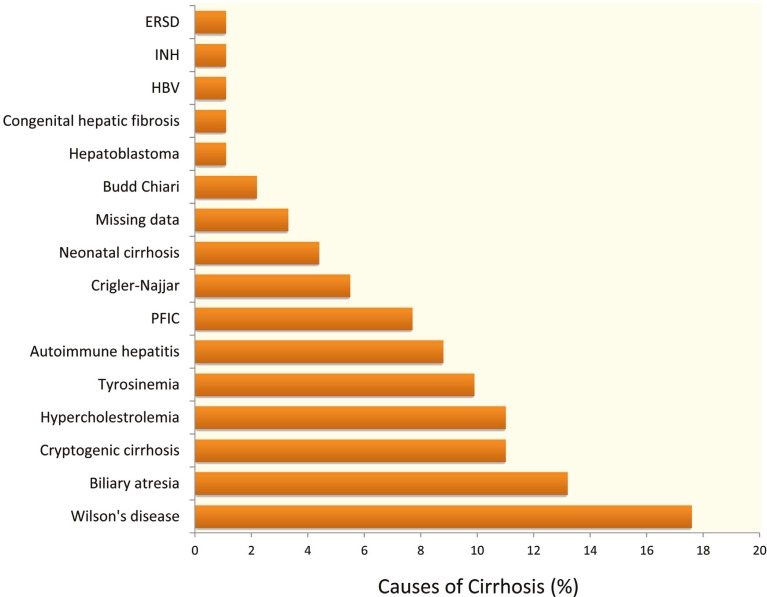
Distribution of causes of cirrhosis among studied pediatric liver transplant recipients.

Wilson’s disease was the cause of cirrhosis in 16 patients and the most common cause of liver failure in our patients. It was followed by biliary atresia (n=12), hypercholesterolemia (n=10), and cryptogenic cirrhosis (n=10) (Fig 1). The mean±SD PELD score of the patients when they were put on the waiting list was 18.53±12.32 (range: 1–60). Their mean±SD CHILD score was 8.01±2.28 (range: 5–13).

Of 91 studied patients, 48 (53%) developed early complications; 38 (42%) developed late complications; and 5 (5%) died after six months of liver transplantation. Fourteen patients were expired within the six months. 

Graft rejection was the most common early complication (n=9). It was followed by convulsion (n=5), and internal bleeding (n= 3). The most common late complication observed was graft rejection (n=12) followed by infection (n=7). 

**Figure 2:**
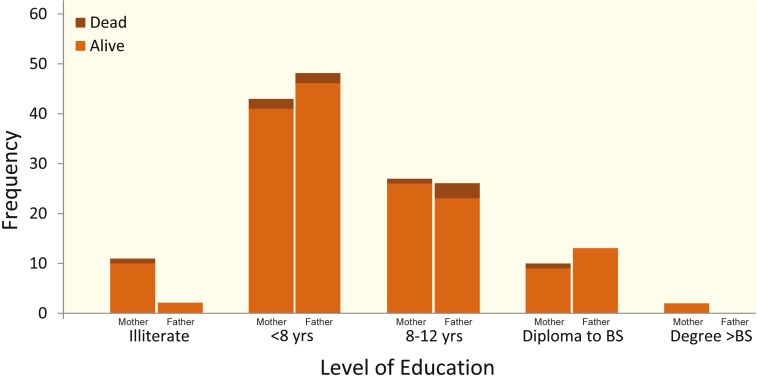
Frequency of death among studied children stratified by parents' level of education

Level of maternal and paternal education as well as the number of dead among studied patients are mentioned in Figure 2. Most of the early and late complications occurred among patients whose parents were less than eight years schooling (Tables 1). Mothers of three patients who died were schooled less than eight years. Fathers of five expired patients were schooled up to diploma, although none of them were illiterate. 

**Table 1:** Level of parents' education and the frequency of post-transplantation complications in recipients

	Parents' education level				
	Illitrate	<8 yrs	8-12 yrs	Diploma to bachelor degree	>Bachelor degree
Father's education					
Early complication	1	26	12	9	1
Late complications	2	20	14	2	0
Mother's education					
Early complication	5	24	12	7	0
Late complications	7	18	9	4	0

## DISCUSSION

Liver transplantation is the last option for the treatment of children with liver failure. Complications and mortality of the procedure are higher among children, especially among younger children. In addition, shortage of organ and high cost of the procedure make the transplant centers to choose the best recipients with acceptable mortality and morbidity [1, 4]. 

Many studies have so far been conducted on pediatric liver transplantation to determine predictive factors influencing the morbidity and mortality of the procedure. For instance, Nairs, *et al *[3], studied the graft and patients survival in various races and concluded that survival in the Black and Asians is less than the Caucasian patients. Schweizer, *et al* [5], and De Geests, *et al* [6], showed that patient compliance is the most common cause of graft loss in both renal and liver transplant recipients. 

Dobbles, *et al* [2], showed that higher education, lower received practical and informational support, and lower score on personality trait consciousness were independent predictors of non-adherence of transplant recipients with medication. Being religious and believing in God are associated with better survival after transplantation [7]. Education level has been shown to have an important impact on renal transplant outcome [8]. Lower income and lack of private insurance were also found to be associated with graft loss [8, 9]. Lack of partnership (*ie*, unmarried or not living together in a stable relationship) is a significant predictor of late graft loss too [2]. Another study showed that heart transplanted patients with empathetic and supportive partner had better surgical and post-transplantation outcomes compared to those without such active relational involvement [10].

Several studies have so far been surveyed the role of education as a predictor of patient and graft survival. The results are, however, quite controversial ranging from negative to positive impact on patient and graft survival [2, 11, 12]. 

All of the above studies have been performed on adult patients. Mostly being cared by their parents, children are a special group in transplantation field. Pediatric patients have almost no role in medication taking and seeking treatment. Therefore, we tested the hypothesis if parents’ education level has any impacts on late mortality and complications in pediatrics transplant recipients and found that most complications (early or late) occur in children whose parents were schooled less than eight years.

Five patients were expired after six months of liver transplantation. Mothers of three expired patients were schooled less than eight years. Fathers of five expired patients were schooled up to diploma; none of them were illiterate.

Multivariate analysis of all groups showed that paternal education (p=0.024) is an independent predictor for the late post-transplantation complications. Educational level of mothers had no significant effect (p=0.45) on the late post-transplantation complication rate. Neither maternal (p=0.59) nor paternal (p=0.607) education had impact on the late post-transplantation mortality.

Many factors influence late post-transplantation mortality and complications of pediatric liver transplant recipients. Post-transplant medication adherence and regular follow-up visits are two examples. We showed that only paternal education level is associated with late post-transplantation complications in pediatric liver transplant recipients. Maternal education is not statistically associated with late post-transplantation mortality and complications. 

Appropriate outcome of pediatric liver transplantation in Namazee hospital observed in this study would be due to the following reasons: a) In this center, post-transplantation follow up of patients is performed “actively” by the staff of coordination office of organ transplantation; *ie*, a coordinator staff contacts the patient or patient caregiver. She or he follows whether the patient has desirable medication adherence, follow up visits by hepatologist, routine work ups, *etc*. The coordinator also notifies the consequences of medication non adherence; b) In selecting candidates of liver transplantation at our center, presence of a perseverant caregiver is always an advantage. Therefore, the impact of education level can be masked; c) the vast majority of recipients in our center are from urban regions, consequently, the level of education is basically higher in our study.

In conclusion, our results indicated that recipient’s mother education in pediatric liver transplantation should not be considered a contraindication of pediatric liver transplantation. Recipient’s father education had a significant impact on the late post-transplantation complications rate.

## References

[1] Rabkin JM, de La Melena V, Orloff SL (2001). Late mortality after orthotopic liver transplantation. Am j Surg.

[2] Dobbels F, Vanhaecke J, Dupont L (2009). Pretransplant predictors of posttransplant adherence and clinical outcome: an evidence base for pretransplant psychosocial screening. Transplantation.

[3] Nair S, Eustace J, Thuluvath PJ (2002). Effect of race on outcome of orthotopic liver transplantation: a cohort study. Lancet.

[4] Dobbels F, Vanhaecke J, Desmyttere A (2005). Prevalence and correlates of self-reported pretransplant nonadherence with medication in heart, liver, and lung transplant candidates. Transplantation.

[5] Schweizer RT, Rovelli M, Palmeri D (1990). Noncompliance in organ transplant recipients. Transplanation.

[6] De Geest S, Borgermans L, Gemoets H (1995). Incidence, determinants, and consequences of subclinical noncompliance with immunosuppressive therapy in renal transplant recipients. Transplantation.

[7] Bonaguidi F, Michelassi C, Filipponi F, Rovai D (2010). Religiosity associated with prolonged survival in liver transplant pecepients. Liver Transplantation.

[8] Butkus DE, Dottes AL, Meydrech EF, Barber WH (2001). Effect of poverty and other socioeconomic variables on renal allograft survival. Transplantation.

[9] Yoo HY, Thuluvath PJ (2004). Outcome of liver transplantation in adult recipients: Influence of Neighborhood income, education, and Insurance. Liver transplantation.

[10] Bunzel B, Wollenek G (1994). Heart transplantation: are there psychosocial predictors for clinical success of surgery?. Thorac Cardiovasc Surg.

[11] Bunzel B, Laederach-Hofmann K (2000). Solid organ transplantation: Are there predictors for posttrnsplant noncompliance? A literature overview. Transplantation.

[12] Parra AV, Rodrigues V, Cancella S (2008). Impact of socioeconomic status on outcome of a Brazilian heart transplant recipients cohort. Int J cardiol.

